# Qianshi Mixture Treats Diabetic Nephropathy by Regulating Lipid Metabolism Reprogramming and Inhibiting Oxidative Stress Damage

**DOI:** 10.1111/jcmm.70628

**Published:** 2025-05-26

**Authors:** Jian Liu, Boning Liu, Enzhi Fan, Han Zhang, Zhonglai Yin, Shuquan Lv, Weibo Wen, Feitian Min, Zhongyong Zhang, Huantian Cui

**Affiliations:** ^1^ Department of Chinese Medicine Cangzhou Hospital of Integrated Traditional Chinese Medicine and Western Medicine of Hebei Cangzhou China; ^2^ Department of Graduate School Hebei University of Traditional Chinese Medicine Shijiazhuang China; ^3^ Department of Graduate School Guangxi University of Chinese Medicine Nanning China; ^4^ Department of Graduate School Shanxi University of Chinese Medicine Taiyuan China; ^5^ Department of Chinese Medicine Cangzhou Central Hospital Cangzhou China; ^6^ Department of Endocrinology Cangzhou Hospital of Integrated Traditional Chinese Medicine and Western Medicine of Hebei Cangzhou China; ^7^ Department of First Clinical Medicine Yunnan University of Chinese Medicine Kunming China

**Keywords:** diabetic nephropathy, lipid metabolism reprogramming, oxidative stress, pharmacological mechanism, Qianshi mixture

## Abstract

Diabetic nephropathy (DN) is a major complication of diabetes that can advance to end‐stage renal disease, posing a substantial health risk. The Qianshi Mixture (QSM) has shown therapeutic potential for DN; however, its pharmacological mechanisms remain insufficiently understood. We developed a DN model in mice and administered QSM as an intervention. To assess QSM's therapeutic effects, we measured the renal‐function‐related biochemical indicators and examined kidney pathological changes. We then applied transcriptomics and non‐targeted metabolomics to explore QSM's impact on gene expression and metabolic products within DN mice renal tissues. Based on our multi‐omics analysis, the effect of QSM on lipid‐metabolism‐related protein expression was confirmed by western blot in kidney tissue. Additionally, we evaluated the antioxidant and anti‐apoptotic properties of QSM by measuring oxidative stress indicators. QSM intervention improved hyperglycemia and proteinuria in DN mice. It also reduced key markers of renal dysfunction whilst alleviating pathological changes in kidney tissue. Through transcriptomic and metabolomic analyses, we identified that QSM affected genes and metabolites involved in lipid metabolism pathways. Notably, differentially expressed genes included *Ces2h*, *Ces1f* and *Alox5*, whilst metabolites such as EPA, 9‐Oxo‐ODE and LPC (20:3) were altered. Further validation revealed that QSM increased the protein levels of CES2H and CES1F whilst decreasing the expression of FABP1, CD36, ALOX15 and ALOX5. Additionally, QSM reduced oxidative stress markers. QSM inhibited both oxidative stress and apoptosis in kidney tissues. QSM protects renal tissues in DN, likely through the regulation of lipid metabolism and the mitigation of oxidative stress damage.

## 1 Introduction

Diabetic nephropathy (DN) is the most common diabetes mellitus (DM) microvascular complication [1] and a primary cause of end‐stage renal disease (ESRD) [2] The International Diabetes Federation reports that the worldwide diabetic patients currently reached 573 million, with projections estimating a rise to 784 million by 2045 [3]. Approximately 30% of patients with type 1 DM (T1DM) and 40% of T2DM are at risk of developing DN [4]. Standard treatment for DN primarily targets the regulation of blood glucose, pressure and lipids [5]. Medications, including inhibitors for oral Renin‐Angiotensin System (RAS) and Sodium‐Glucose Cotransporter 2 (SGLT2), as well as non‐steroidal mineralocorticoid receptor antagonists (NS‐MRA), have a significant therapeutic effect regarding managing DN. However, their long‐term use may result in side effects and complications, and they fail to reverse the progression of DN [6]. As a result, there is a pressing need to develop novel, safer and more effective therapeutic strategies.

The pathological mechanisms underlying DN are highly complex, involving metabolic reprogramming, inflammation and oxidative stress [6, 7]. Metabolic reprogramming plays a central role in the onset and progression of DN. The kidney, as a high‐energy‐consuming organ, depends on a balanced energy metabolism system to maintain its normal physiological functions [8]. However, patients with DN exhibit significant abnormalities in glucose and lipid metabolism [9]. Hyperglycemia and hyperlipidemia impair mitochondrial oxidative phosphorylation, leading to an increased metabolic flux through glycolysis to rapidly produce ATP in response to these imbalances [10]. This compensatory shift, known as metabolic reprogramming, represents a mechanism by which cells alter their metabolic pathways to meet the energy demands required for survival and growth [11].

However, prolonged metabolic reprogramming can result in mitochondrial dysfunction, further exacerbating oxidative stress. This damage contributes to the thickening of the basement membrane and hypertrophy of glomeruli and renal tubules [12], which in turn manifests as clinical signs of renal injury, including proteinuria and edema. Additionally, elevated glucose and lipid levels can activate various immune cells, triggering inflammatory responses that intensify renal damage in DN. Correcting metabolic reprogramming has shown potential in slowing the progression of DN [13].

Non‐targeted metabolomics techniques enable the comparison of metabolite levels across different samples, providing insights into the specific manifestations and mechanisms of metabolic reprogramming during the progression of DN. A non‐targeted metabolomics study identified 15 dysregulated metabolites, including amino acids and their derivatives, monosaccharides, organic acids and uremic toxins, which could serve as potential biomarkers for advanced DN. These biomarkers may assist in staging and tailoring treatments for the condition [14].

Since metabolite expression is regulated by various metabolic enzymes, integrating transcriptomics with non‐targeted metabolomics allows for a deeper understanding of gene expression related to enzymes that influence metabolic changes. This combined approach also facilitates the analysis of biological processes associated with these metabolic alterations. Research has shown that, in the renal tissues of DN patients, the expression of genes encoding transport proteins or receptors involved in fatty acid and cholesterol uptake is elevated, whilst the expression of genes responsible for cholesterol efflux is reduced, resulting in abnormal lipid metabolite levels [15]. Additionally, alterations in amino acid metabolism, particularly in genes related to tryptophan metabolism and their metabolites, are strongly associated with DN progression [16, 17]. Thus, the integration of transcriptomics and non‐targeted metabolomics provides a comprehensive understanding of how drug interventions may influence metabolic reprogramming in DN.

Traditional Chinese Medicine (TCM) has long been used to treat diabetes and its complications [18, 19, 20]. Clinical studies have demonstrated the effectiveness of various TCM formulations, such as Tongluo capsules [21], Yiqi Huayu Jiangzhuo formula [22] and Zishen Tongluo granules [23], in alleviating renal damage associated with DN. Qianshi Mixture (QSM), a TCM formulation containing 10 herbal ingredients—including *Euryale ferox* Salisb., *Atractylodes macrocephala* Koidz., *Poria cocos* (Schw.) Wolf, *Dioscorea opposita* Thunb., *Cuscuta australis* R.Br., *Rosa laevigata Michx*., *Polygonatum kingianum* Coll. et Hemsl., *Lilium lancifolium* Thunb., *Eriobotrya japonica* (Thunb.) Lindl. and *Codonopsis pilosula* (Franch.) Nannf. has shown potential in treating several chronic kidney diseases, including DN [24]. However, the precise mechanisms by which QSM exerts its effects in DN remain unknown and require further exploration. In this study, we first assessed the therapeutic effects of QSM on DN in mice. Next, we investigated the mechanisms by which QSM regulates metabolic reprogramming in DN by integrating transcriptomic and non‐targeted metabolomic analyses. Finally, we validated the impact of QSM on factors related to fatty acid metabolism and assessed its antioxidant and anti‐apoptotic effects.

## 2 Materials and Methods

### 2.1 Animals and Reagents

We procured 60 healthy, SPF‐grade male C57BL/6 mice, aged 6 to 8 weeks and weighing an average of 21 ± 1 g, from Beijing Huafukang Biotechnology Co. Ltd. (Licence No: SCXK (Beijing) 2019–0008). The mice were housed in groups of five per cage in accordance with the ‘Guide for the Care and Use of Laboratory Animals’ from the National Institutes of Health. Environmental conditions were carefully maintained at a temperature of 24°C ± 2°C and a relative humidity of 55% ± 5%, with bedding kept dry. A 12‐h light/dark cycle was applied, and the mice had free access to food and water. After a 1‐week acclimatisation period, we proceeded with the experiments. Details of reagents and other materials used in the experiments are available in the Supporting Information S1 (Reagent).

### 2.2 Preparation of Qianshi Mixture

Following the QSM recipe, the specified amounts of Chinese medicinal herbs were weighed: 30 g of *Euryale ferox* Salisb., 12 g of *Atractylodes macrocephala* Koidz., 12 g of *Poria cocos* (Schw.) Wolf, 15 g of *Dioscorea opposita* Thunb., 24 g of *Cuscuta australis* R.Br., 24 g of *Rosa laevigata Michx*., 24 g of *Polygonatum kingianum* Coll. et Hemsl., 18 g of *Lilium lancifolium* Thunb., 9 g of *Eriobotrya japonica* (Thunb.) Lindl. and 9 g of *Codonopsis pilosula* (Franch.) Nannf. These herbs, procured from the traditional Chinese medicine pharmacy of the Cangzhou Hospital of Integrated Traditional Chinese Medicine and Western Medicine of Hebei Province, were combined, soaked in eight times the amount of pure water for 2 h, boiled over high heat, and simmered over low heat for 0.5 h, and the medicinal liquid was collected. After filtering, the medicinal liquid underwent centrifugation at 1500 rpm for 10 min, followed by filtration. The combined solutions were concentrated to 8 g crude herb/mL and stored in a 4°C refrigerator for future use.

### 2.3 Modelling, Grouping and Administration

Following the modelling method described in the literature [25], we randomly assigned the mice into two groups. The Control group (*n* = 10) received a normal diet, whilst the remaining 50 mice were fed a high‐fat, high‐sugar diet consisting of 21% fat, 34% sucrose, 0.15% cholesterol and 44.85% standard feed for 8 weeks before modelling. The modelling procedure began with a 15‐h fast, with free access to water. We then administered an intraperitoneal injection of streptozotocin (STZ) at a dose of 30 mg/kg to induce type 2 DM (T2DM). The Control group received an equivalent volume of 1% sodium citrate buffer via intraperitoneal injection. After 72 h, we measured blood glucose levels using a threshold of ≥ 16.7 mmol/L to confirm the onset of T2DM. Mice were maintained on their respective diets until confirmation of the model. We performed weekly assessments, including 24‐h urinary protein quantification (24 h‐UTP). A random blood glucose level of ≥ 16.7 mmol/L and a 24 h‐UTP of ≥ 20 mg were set as the criteria for confirming DN modelling. Two weeks post‐STZ injection, the modelled mice met the established criteria for DN.

After successfully establishing the DN model, we randomly assigned the mice into five groups: Model group (DN), Irbesartan group (IRBE), low‐dose QSM group (QSML), medium‐dose QSM group (QSMM) and high‐dose QSM group (QSMH), with 10 mice per group. The Control and DN groups each received 0.2 mL/day of physiological saline via gavage, whilst the IRBE group was each administered IRBE at 40 mg/day [26]. The low, medium and high‐dose QSM groups were given 11.5, 23 and 46 mg/kg/day, respectively, with the medium dose corresponding to the clinical equivalent dose for humans. Gavage treatment continued for 8 weeks. During this period, we recorded blood glucose levels and body weights weekly. After 8 weeks of treatment, the mice were fasted for 12 h and weighed, and 24‐h urine samples were collected using metabolic cages. Blood samples were drawn via the medial canthus. Following euthanasia, we collected the left kidney and fixed it in 4% paraformaldehyde and rapidly froze the right kidney in liquid nitrogen, which was then stored at −80°C for further analysis.

### 2.4 Biochemical Indicator Testing

Collected 24‐h urine samples were centrifuged (4000 rpm, 10 min), and their supernatant was analysed for the 24 h‐UTP levels following the assay kit instructions. Blood samples were centrifuged for 10 min at 3500 rpm, where the obtained serum was then analysed for creatinine (Cr) and blood urea nitrogen (BUN) levels by using assay kits. Frozen kidney tissue was homogenised with physiological saline (1:9). After 15 min centrifugation at 4000 rpm, the collected homogenate was used to measure superoxide dismutase (SOD) and glutathione peroxidase (GSH‐Px) activity, as well as 4‐hydroxynonenal (4‐HNE), reactive oxygen species (ROS) and malondialdehyde (MDA) levels using appropriate biochemical assay kits. We also assessed the total protein concentration in the tissue homogenate for sample normalisation, with procedures following the specific instructions of the assay kits.

### 2.5 Histological Staining

Tissue samples were fixed in 4% paraformaldehyde, dehydrated and embedded in paraffin. The blocks were then sectioned into 4 μm thick slices. Following sectioning, the tissue underwent haematoxylin and eosin (HE), Masson's trichrome, periodic acid‐Schiff (PAS) and terminal uridine nick‐end labeling (TUNEL) staining. For HE staining, the pathological morphology of renal tissue was scored based on the degree of glomerular hypertrophy and mesangial matrix proliferation. The scoring criteria were as follows: 0 (none), 1 (≤ 10%), 2 (11%–25%), 3 (26%–45%), 4 (46%–75%) and 5 (≥ 76%) as previously described. For Masson's staining, the extent of fibrosis in the renal tissue was assessed by evaluating the blue‐stained collagen fibres. The calculation method was as follows: positive area = positive area/total tissue area. For PAS staining, the glycogen deposition in the renal tissue was assessed based on the intensity of the magenta coloration. The calculation method was as follows: Positive area = Positive area/Total tissue area. After mounting, the tissue sections were carefully examined under a microscope, and images were captured for subsequent analysis. TUNEL staining was performed to detect apoptosis in the renal tissue, with positive staining areas quantified using Image J software.

### 2.6 Transcriptomics

After 8 weeks of QSM intervention in DN mice, kidney tissues were collected for transcriptomic analysis. The analytical methods were adapted from previous studies [27]. In brief, total RNA was extracted from the kidney tissues, and the purity, concentration and integrity of the RNA were assessed. Only samples meeting the quality standards were used for subsequent library preparation and sequencing, which was performed using the Illumina platform. Differentially expressed genes (DEGs) were identified by comparing the DN group with the Control group and the QSM group with the DN group using ESeq2 software. The selection criteria for DEGs were set at |Log_2_(Fold Change)| > 1 and *p*_adj_ < 0.05. Gene Ontology (GO) enrichment analysis was then conducted on the identified DEGs.

### 2.7 Non‐Targeted Metabolomics

To investigate metabolite alterations in renal tissue, we employed non‐targeted metabolomics. Kidney tissue samples (100 mg from each group) were homogenised in cold water and centrifuged to collect the supernatant. The supernatant was then dried under nitrogen, reconstituted in methanol containing internal standards and vortexed. Afterward, the mixture was centrifuged at 12,000 rpm for 15 min, and the resulting supernatant was transferred to an injection vial. External standard solutions were added to the sample for UPLC‐MS/MS analysis. An equal volume of supernatant from all samples was pooled to create a quality control (QC) sample. The metabolomics protocols were adapted from previous studies [28].

### 2.8 Western Blot

Total protein was extracted from 50 mg of kidney tissue and quantified using the bicinchoninic acid (BCA) method. After loading buffer was added, we denatured the proteins in a metal bath (99°C, 5 min). After SDS‐PAGE separation, the proteins were transferred to a PVDF membrane using the wet transfer method. To block non‐specific binding, we incubated the membrane with a 5% non‐fat milk solution for 2 h. Next, we incubated the membrane with primary antibodies against the protein of interest (4°C, overnight). After three washes with TBST, we applied an HRP‐conjugated secondary antibody (1:4000 dilution) and incubated it for 2 h at room temperature. Following three additional washes, we added enhanced chemiluminescence (ECL) reagent for protein detection. We visualised the protein bands with a gel imaging system that was fully automated, and the grayscale values of the bands were analysed statistically using ImageJ software.

### 2.9 Statistical Analysis

We analysed the experimental results using SPSS Statistics 22.0 software. Data are expressed as mean ± SD. To compare two groups, we used a two‐tailed Student's t‐test. For comparisons amongst multiple groups, we performed a one‐way analysis of variance (ANOVA) followed by Tukey's post hoc test. *p* < 0.05 and *p* < 0.01 represent significant difference and highly significant difference, respectively.

## 3 Results

### 3.1 QSM Exhibits Therapeutic Effects on DN Mice

**FIGURE 1:**
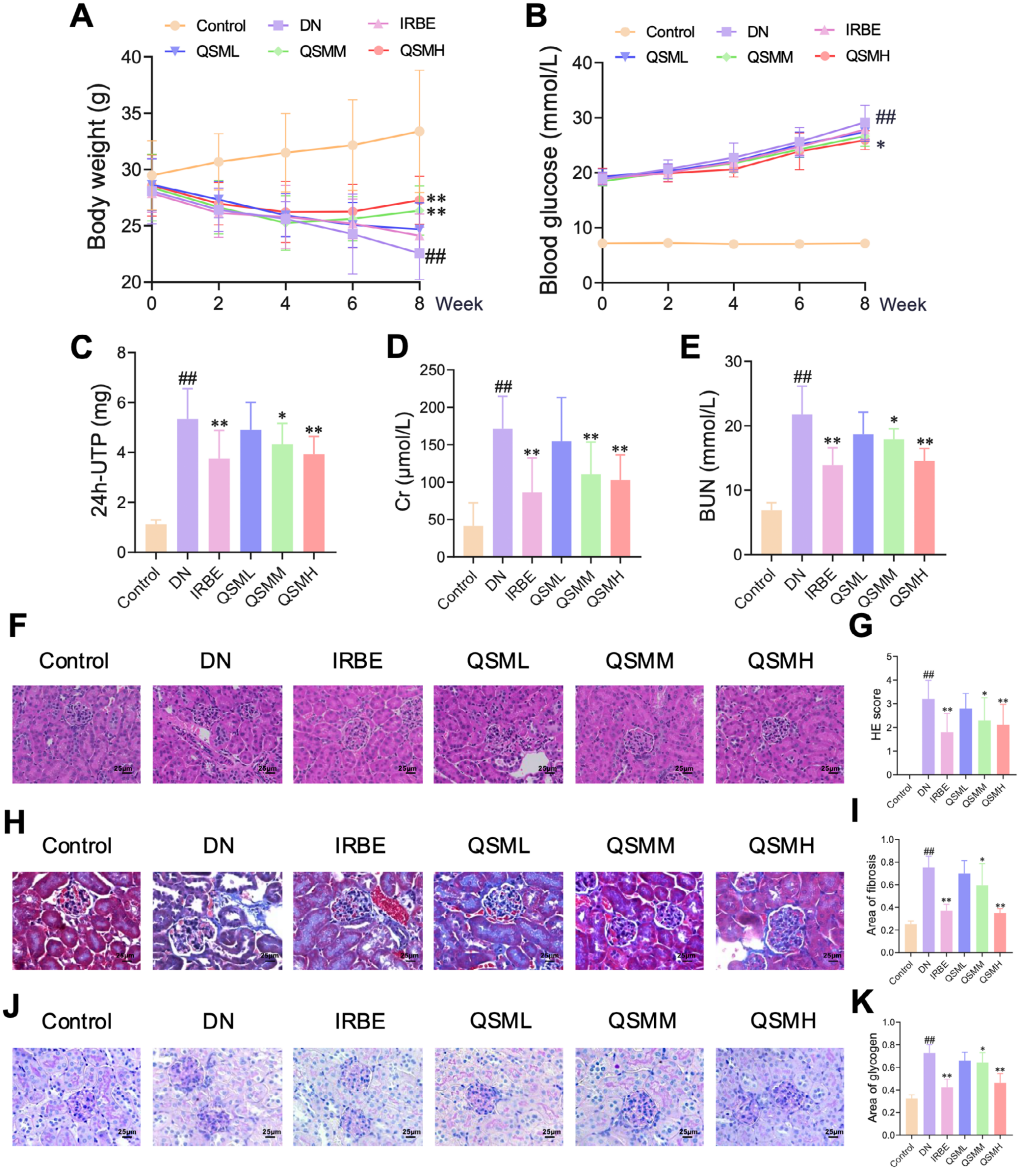
QSM alleviates renal tissue damage in DN mice. A DN mouse model was established and subjected to QSM intervention. (A, B) QSM treatment significantly improves body weight loss (A) and hyperglycemic status (B) in DN mice. (C–E) QSM intervention leads to decreased levels of 24‐h urinary total protein (C), creatinine (D) and blood urea nitrogen (E). (F–K) Histopathological staining results indicating that QSM intervention mitigates glomerular hypertrophy, mesangial matrix proliferation (F, G), reduced renal tissue fibrosis (H, I) and decreased glycogen deposition in renal tissue (J, K). Data are presented as mean ± SD, *n* = 10 per group. Statistical significance is indicated as ^##^ *p* < 0.01 compared to the Control group; *p < 0.05, **p < 0.01 compared to the DN group.

After 8 weeks of treatment, body weight measurements revealed a significant reduction in the DN group compared to the Control group. In contrast, both the QSM and IRBE groups showed varying degrees of weight gain relative to the DN group (Figure 1A). Biochemical analyses indicated that, compared to the Control group, mice in the DN group exhibited markedly elevated levels of blood glucose, 24 h‐UTP, Cr and BUN in serum. In contrast, the IRBE group showed reduced serum levels of 24 h‐UTP, Cr and BUN compared to the DN group, whilst the QSM group also demonstrated varying degrees of reduction in these parameters (Figure 1B–E). HE staining revealed glomerular hypertrophy and mesangial matrix proliferation in the DN group, whereas the IRBE, QSML, QSMM and QSMH groups exhibited varying degrees of renal improvement (Figure 1F,G). Masson staining highlighted an increased presence of blue areas in the DN group, indicative of fibrotic changes in renal tissue, whilst the IRBE, QSML, QSMM and QSMH groups showed reduced collagen fibre distribution (Figure 1H,I). PAS staining displayed deeper magenta coloration, suggesting greater glycogen accumulation in the kidneys of DN group mice. In contrast, the IRBE, QSML, QSMM and QSMH groups demonstrated varying degrees of reduction in glycogen deposition (Figure 1J,K). These results suggest that QSM has a therapeutic effect on DN, with efficacy exhibiting a dose‐dependent relationship, particularly in the QSMH group, which showed results comparable to those of positive drug interventions. Therefore, we selected the high‐dose QSM for subsequent mechanistic studies.

### 3.2 The Impact of QSM on the Transcriptome of Renal Tissue in DN Mice

**FIGURE 2:**
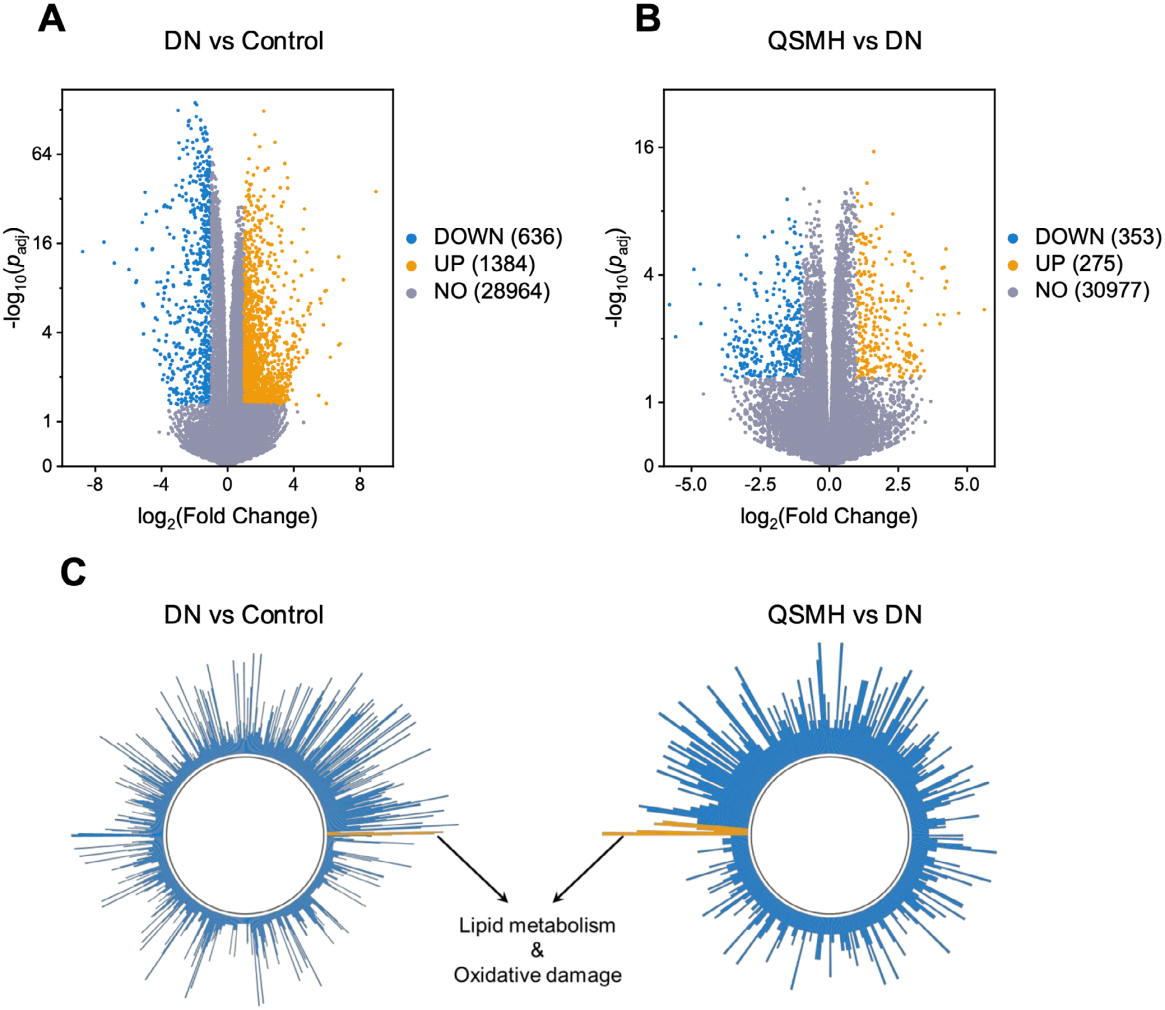
QSM intervene in the progression of DN by modulating lipid metabolism and oxidative damage‐related pathways. |Log_2_(Fold Change)| > 1 and *p*_adj_ < 0.05 were used for identification of DEGs between DN and Control and also between QSMH and DN mice. (A, B) Volcano plots visualising DEGs. (C) GO results revealing that the detected DEGs are significantly enriched in lipid metabolism‐ and oxidative damage‐related pathways. *n* = 6 per group.

We performed transcriptomic sequencing on the Control, DN and QSMH groups, using the criteria |Log_2_(Fold Change)| > 1 and *p*_adj_ < 0.05 to identify DEGs across the groups. The volcano plot shows that, compared to the Control group, the DN group exhibited upregulation of 1384 genes and downregulation of 636 genes (Figure 2A). In contrast, the QSMH group showed upregulation of 275 genes and downregulation of 353 genes relative to the DN group (Figure 2B). GO enrichment analysis of these DEGs identified 926 enriched pathways for the comparison between DN and Control and 429 enriched pathways for the comparison between QSMH and DN. Amongst these pathways, we identified those most significantly impacted, which were primarily related to lipid metabolism and oxidative damage (Figure 2C). These findings suggest that QSM may intervene in the progression of DN through modulation of these two pathways.

### 3.3 The Impact of QSM on the Metabolome of Renal Tissue in DN Mice

**FIGURE 3:**
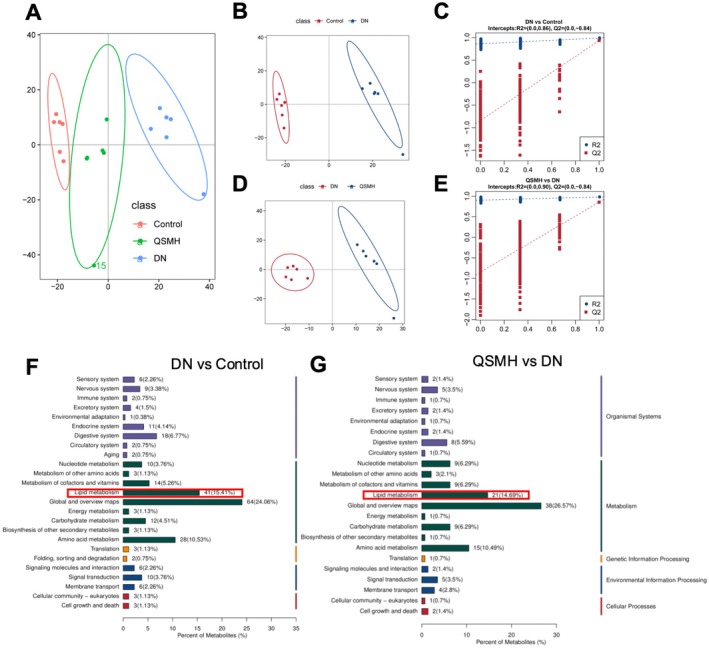
QSM ameliorates DN by modulating lipid metabolism. Non‐targeted metabolomic analysis on Control, DN and QSMH groups. (A) PCA results indicating significant differences in metabolite levels within renal tissues amongst Control, DN and QSMH groups; (B–E) PLS‐DA validation results demonstrating strong fitting and predictive capabilities for DN versus Control (B, C) and QSMH versus DN (D, E). (F, G) KEGG pathway enrichment analysis for DN and Control groups (F), as well as for QSMH and DN groups (G). *n* = 6 per group.

We performed principal component analysis (PCA) on the non‐targeted metabolomics data of renal tissues from the Control, DN and QSMH groups. The results revealed significant differences in the metabolic profiles amongst these groups (Figure 3A). To further investigate these differences, we established a predictive model and validated it using partial least squares‐discriminant analysis (PLS‐DA). The *R* ^2^ value for the DN versus Control comparison was 0.86, and the *Q* ^2^ value was −0.84. For the QSMH versus DN comparison, the *R* ^2^ value was 0.9, and the *Q* ^2^ value was −0.84. These values indicate that the statistical model provides a good fit and strong predictive capability (Figure 3B–E). Next, we identified differential metabolites between the groups using the criteria *p* < 0.05, VIP > 1 and FC > 1.5 or < 0.67. KEGG Classification analysis of the identified metabolites revealed key pathways for both DN versus Control and QSMH versus DN, including Global and Overview Maps, Lipid Metabolism and Amino Acid Metabolism (Figure 3F,G). Integration of these metabolomic findings with the transcriptomic analysis suggests that lipid metabolism plays a critical role in the way QSM ameliorates DN.

### 3.4 QSM Intervention Regulates Lipid Metabolism Reprogramming in DN Mice

**FIGURE 4:**
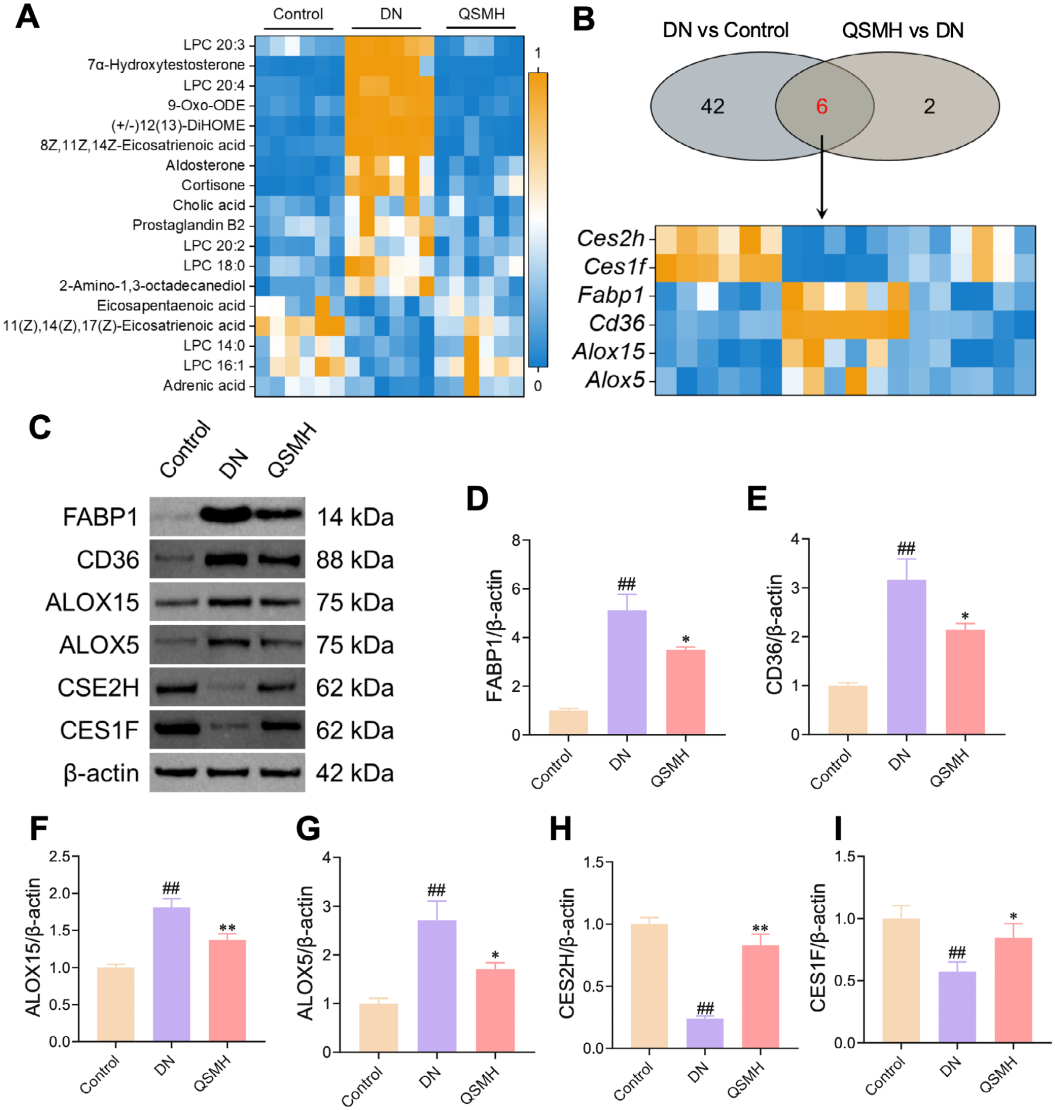
QSM intervention regulates lipid metabolism reprogramming in DN mice. (A) Heatmap of lipid metabolism‐related products revealing that QSMH intervention downregulates levels of lipid metabolites, such as LPC (20:3), 7α‐hydroxy testosterone, LPC (20:4) and 9‐Oxo‐ODE. (B) Heatmap of lipid metabolism‐related gene expression indicating that QSMH intervention markedly upregulates expression of *Ces1f* and *Ces2h* genes whilst downregulating expression of *Fabp1*, *Cd36*, *Alox15* and *Alox5* genes. (C, I) Western blot results demonstrating that following QSM intervention, expression of FABP1 (C, D), CD36 (C, E), ALOX15 (C, F) and ALOX5 (C, G) significantly reduced, whilst expression of CES1F (C, H) and CES2H (C, I) significantly increased. *n* = 6 per group for A, *n* = 6 per group for B, *n* = 3 per group for C–I.

Based on the transcriptomic and metabolomic findings, the regulation of lipid metabolism appears to be a critical mechanism through which QSM improves DN. To investigate this, we first examined the impact of QSM intervention on lipid metabolites. The results showed that OSMH intervention upregulated the levels of eicosapentaenoic acid (EPA) and adrenic acid, whilst downregulating LPC (20:3), 7α‐hydroxy testosterone, LPC (20:4) and 9‐0xo‐0DE levels (Figure 4A).

Next, we analysed lipid metabolism‐related genes identified through transcriptomics and found that QSM intervention affected the expression of *Carboxylesterase 2H (Ces2h)*, *Carboxylesterase 1F (Ces1f)*, *fatty acid‐binding protein 1 (Fabp1)*, *CD36 molecule (Cd36)*, *Arachidonate 5‐lipoxygenase (Alox15)* and *Arachidonate 5‐lipoxygenase (Alox5)* genes (Figure 4B). To confirm these findings at the protein level, we performed Western blot analysis to assess the expression of CES2H, CES1F, FABP1, CD36, ALOX15 and ALOX5 in renal tissue. Compared to the Control group, the DN group showed significant upregulation of FABP1, CD36, ALOX15 and ALOX5 protein levels, whilst the expression of CES1F and CES2H was notably downregulated. In contrast, QSMH intervention significantly reduced the expression of FABP1, CD36, ALOX15 and ALOX5 proteins, whilst upregulating CES1F and CES2H protein expression (Figure 4C–I). These findings further support the idea that QSM improves DN by modulating lipid metabolism.

### 3.5 QSM Intervention Inhibits Oxidative Damage in DN Mice

**FIGURE 5:**
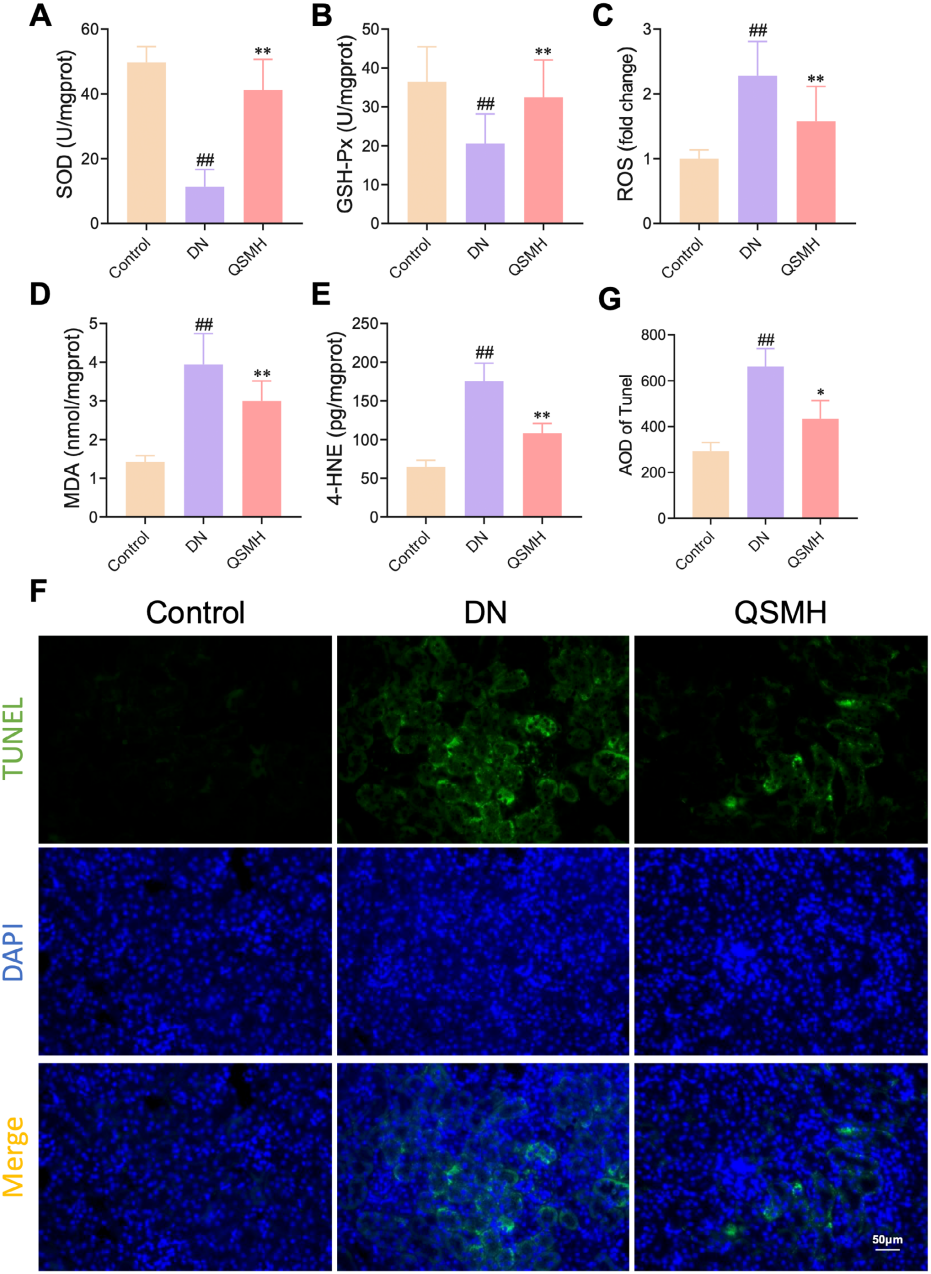
QSM inhibits oxidative stress and apoptosis in DN mice. (A–E) QSM intervention significantly enhances SOD (A) and GSH‐Px (B) activity whilst reducing ROS (C), MDA (D) and 4‐HNE (E) levels. (F, G) QSM intervention reduces TUNEL‐positive areas in renal tissue. *n* = 10 per group. Statistical significance is indicated as ##*p* < 0.01 compared to the Control group; *p < 0.05, **p < 0.01 compared to the DN group.

Lipid metabolism and oxidative stress are closely linked, with oxidative stress playing a crucial role in the progression of DN‐related renal injury. To explore this relationship, we assessed the impact of QSM intervention on oxidative stress and tissue damage in renal tissues. The analysis of oxidative stress markers revealed a decrease in the activities of SOD and GSH‐Px in the renal tissues of DN mice, along with an increase in ROS, 4‐HNE and MDA levels. However, QSMH intervention effectively reversed these oxidative stress markers (Figure 5A–E). Additionally, TUNEL staining showed a significant increase in renal cell apoptosis in the DN group, which was markedly reduced following QSMH treatment (Figure 5F,G). These results suggest that QSM mitigates oxidative stress and apoptosis in renal tissues.

## 4 Discussion

DN is a severe complication that significantly impacts the health of diabetic patients, yet its pathogenesis remains incompletely understood. This gap in knowledge limits the development of effective clinical treatment strategies for DN. QSM, a TCM commonly used in clinical practice for treating chronic nephritis [29] has not been thoroughly investigated for its specific pharmacological mechanisms and requires further research. In this study, we established a DN mouse model using a high‐fat, high‐sugar diet combined with STZ intraperitoneal injection, a widely recognised method in DN research [30]. As expected, the DN group exhibited hyperglycemia and impaired renal function. Pathological staining revealed significant histological damage to the renal tissue, including collagen fibre and glycogen deposition, which are characteristic features of DN, confirming successful induction of the disease in the mice. Following QSM intervention, the mice showed varying degrees of blood glucose reduction, and several biochemical indicators improved. Pathological examinations further demonstrated that QSM had a positive impact on the renal tissue alterations in DN mice. To evaluate the therapeutic potential of QSM, we included IRBE as a positive control drug, known for its established efficacy in clinical practice [31]. Our results showed no significant differences between the QSMH and IRBE groups in terms of improving blood glucose levels and renal function. These findings suggest that QSM effectively targets DN and may serve as a promising alternative to IRBE for treating the disease.

The integration of transcriptomics and metabolomics to investigate the mechanisms by which QSM ameliorates DN has revealed that QSM can mitigate renal tissue damage by regulating lipid metabolism, thereby exerting therapeutic effects on DN. Previous studies have highlighted the critical role of lipid metabolism in the onset and progression of DN [32]. Our findings from both transcriptomic and metabolomic analyses show that QSM intervention upregulated the expression of the *Ces2h* and *Ces1f* genes, whilst downregulating the expression of *Fabp1*, *Cd36*, *Alox15* and *Alox5* genes. Moreover, QSM treatment increased the levels of Polyunsaturated fatty acids (such as EPA), whilst decreasing the levels of LPC (20:3) and 9‐Oxo‐ODE. Further experimental validation confirmed that QSM's effect on the protein levels of CES2H, CES1F, FABP1, CD36, ALOX15 and ALOX5 aligned with the transcriptomic data. Research has shown that CD36 and FABP1 are critical for the uptake and transport of fatty acids [33]. CD36, a multifunctional transmembrane protein, facilitates the binding of long‐chain fatty acids, oxidised lipids, phospholipids and advanced glycation end products. It plays a pivotal role in lipid accumulation, lipotoxic reprogramming and renal fibrosis [34]. Notably, CD36 expression significantly increases in DN and contributes to apoptotic mechanisms [35]. Similarly, FABP1, a fatty acid‐binding protein, aids in the transport and metabolism of fatty acids [36] and is closely associated with the changes in metabolites such as LPC (20:3) [37]. In diabetic patients, urinary FABP1 levels are significantly elevated, positioning it as a potential biomarker for the early detection and prediction of DN progression [38]. CES2H and CES1F are two key carboxylesterases that hydrolyse endogenous esters, such as lyso‐phosphatidylcholine (LPC), playing a crucial role in lipid metabolism [39, 40, 41, 42]. LPC is cytotoxic and can induce oxidative stress and apoptosis. Consequently, QSM may protect the kidneys by upregulating CES2H and CES1F to enhance LPC hydrolysis. Additionally, the enzymes ALOX15 and ALOX5 catalyse the oxidation of polyunsaturated fatty acids, including EPA, and are closely associated with inflammatory responses and oxidative stress [43]. These enzymes may influence the production of oxidative stress products, such as 9‐oxo‐ODE [44]. Studies have shown that ALOX15 and ALOX5 are expressed in renal cells, with their expression levels increasing as DN progresses [45]. Inhibiting the protein expression of ALOX15 and ALOX5 may offer anti‐fibrotic, anti‐inflammatory and anti‐apoptotic benefits [46]. We hypothesise that QSM may reduce the oxidation of polyunsaturated fatty acids, such as EPA, by inhibiting ALOX15 and ALOX5 activity, thereby reducing oxidative stress‐induced cell damage.

Lipid metabolism reprogramming is closely associated with oxidative stress, a well‐established contributor to proteinuria and renal interstitial fibrosis [47]. To explore this connection further, we examined the effect of QSM on oxidative stress in DN mice. Our results showed that QSM treatment significantly altered oxidative stress markers in the renal tissue of DN mice. Specifically, QSM upregulated the activities of SOD and GSH‐Px, whilst downregulating the levels of MDA, 4‐HNE and ROS. In a hyperglycemic and hyperlipidemic environment, lipid self‐oxidation and oxidative phosphorylation processes generate substantial amounts of ROS. The accumulation of ROS triggers lipid peroxidation in cell membranes [48], leading to increased production of MDA and 4‐HNE, both of which serve as biomarkers of lipid peroxidation [49, 50]. The activities of SOD and GSH‐Px reflect the body's ability to neutralise ROS [51]. However, we also acknowledge that the precise mechanisms underlying the interplay between lipid metabolism regulation and antioxidative effects of QSM require further in‐depth investigation. We will delve deeper into exploring this relationship in future experiments.

Additionally, TUNEL staining was used to assess renal cell apoptosis, revealing that QSM reduced apoptosis levels in renal tissue, further supporting its protective role in preserving renal function. However, whilst TUNEL staining is widely known for detecting apoptosis by identifying DNA fragmentation, it can also be used to observe other types of cell death if DNA damage occurs, including ferroptosis under certain conditions. In our study, we have demonstrated that QSM treatment significantly reduced oxidative stress markers, including ROS, MDA and 4‐HNE, which are closely associated with lipid peroxidation and ferroptosis. These findings suggest that QSM may exert protective effects against ferroptosis by mitigating lipid peroxidation. However, this study did not include specific experiments to directly assess ferroptosis, such as measuring glutathione peroxidase 4 (GPX4) activity and iron content or using ferroptosis‐specific inhibitors or inducers [52, 53]. These represent important directions for future research.

## 5 Conclusion

**FIGURE 6:**
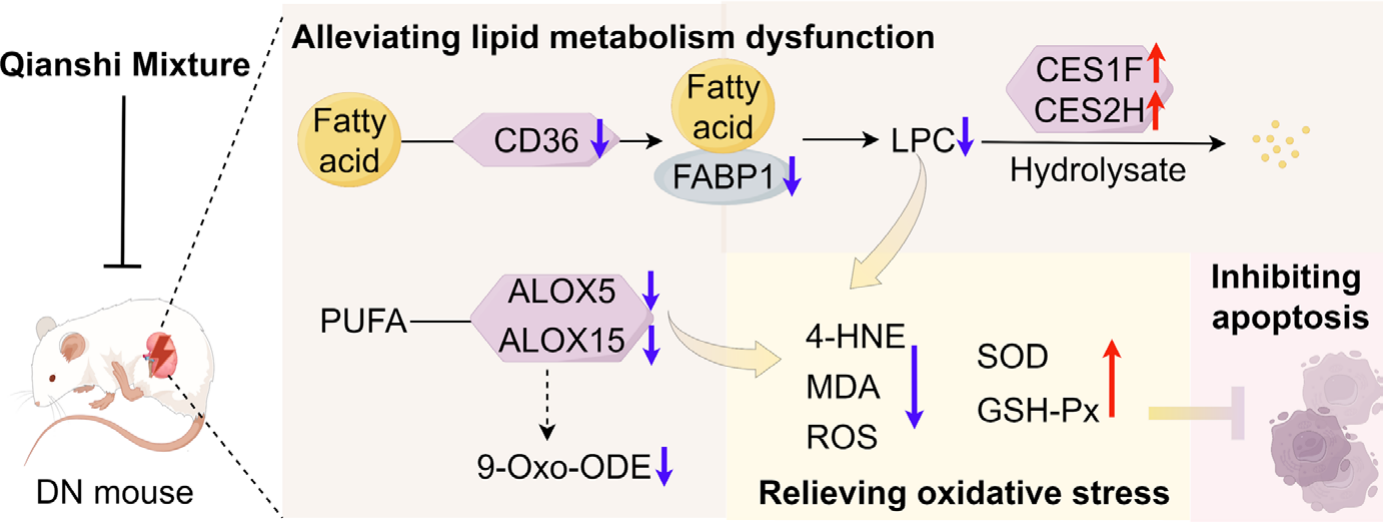
QSM improves renal injury in DN mice via regulation of lipid metabolism reprogramming and inhibition of oxidative stress (by Figdraw).

In summary, QSM shows strong potential as a therapeutic agent for DN. Mechanistic analyses using transcriptomics and non‐targeted metabolomics revealed that QSM mitigates oxidative stress in DN mice by modulating lipid metabolism. This regulation supports the restoration of lipid homeostasis and provides protective effects on renal tissue. Future studies that incorporate lipidomics and other multi‐omics approaches will be crucial for elucidating the precise mechanisms of QSM's action. Such research will help identify specific molecular targets, laying a robust scientific foundation for the further development and clinical application of QSM (Figure 6).

## Author Contributions

**Jian Liu:** conceptualization (equal), funding acquisition (equal), writing – original draft (equal). **Boning Liu:** data curation (equal), writing – original draft (equal). **Enzhi Fan:** data curation (equal), formal analysis (equal). **Han Zhang:** data curation (equal). **Zhonglai Yin:** data curation (equal), investigation (equal). **Shuquan Lv:** data curation (equal), formal analysis (equal), funding acquisition (equal). **Weibo Wen:** investigation (equal). **Feitian Min:** methodology (equal), validation (equal). **Zhongyong Zhang:** conceptualization (equal), software (equal). **Huantian Cui:** conceptualization (equal), writing – review and editing (equal).

## Conflicts of Interest

The authors declare no conflicts of interest.

## Supporting information


Data S1.

## Data Availability

The data that support the findings of this study are available from the corresponding author upon reasonable request.
