# Supplementary material for: Qianshi Mixture Treats Diabetic Nephropathy by Regulating Lipid Metabolism Reprogramming and Inhibiting Oxidative Stress Damage
Source: J Cell Mol Med. 2025 May 26;29(10):e70628. doi: 10.1111/jcmm.70628 (PMC12105581; doi:10.1111/jcmm.70628)
Supplement: Supplementary file 1 — Data S1. [file JCMM-29-e70628-s001.docx]

***Supplementary Material***

**Reagents**

Streptozotocin (S17049), irbesartan (S42406) were purchased from Shanghai yuanye Bio-Technology Co., Ltd.(Shanghai, China). 24h-UTP(C035-2-1), creatinine (Cr, C011-2-1) and blood urea nitrogen (BUN, C013-2-1), SOD(A001-3-2), GSH-Px (A005-1-2), MDA (A003-1-2), 4-HNE ([H268-1-2](http://www.njjcbio.com/products.asp?id=2755)), ROS ([E004-1-1](http://www.njjcbio.com/products.asp?id=819)), HE staining assay kit ([D006-1-1](http://www.njjcbio.com/products.asp?id=476)), Masson ([D026-1-3](http://www.njjcbio.com/products.asp?id=529)), PAS (D004-1-1) were purchased from Nanjing Jiancheng Biological Engineering Institute (Nanjing, China). TUNEL staining assay kit (C1088) was purchased from Beyotime Biotechnology (Shanghai, China). Primary antibodies for CES1F (ab68190, 1/25000), CES2H (ab184957, 1/1000), CD36 (ab252922, 1/1000), ALOX15 (ab244205, 1/1000), ACTB (ab8227, 1/2000) and secondary antibody goat anti-rabbit IgG H&L (ab205718) were purchased from Abcam (Shanghai, China), Primary antibodies for ALOX5 (10021-1-Ig, 1:500) were purchased from Proteintech (Wuhan, China). FABP1 (13368s, 1/1000) were purchased from Cell Signaling Technology (Shanghai, China).
